# Quantification methods of determining brewer’s and pharmaceutical yeast cell viability: accuracy and impact of nanoparticles

**DOI:** 10.1007/s00216-023-04676-w

**Published:** 2023-04-21

**Authors:** Marco Eigenfeld, Leonie Wittmann, Roland Kerpes, Sebastian Schwaminger, Thomas Becker

**Affiliations:** 1 Chair of Brewing and Beverage Technology, Technical University of Munich, TUM School of Life Science, Weihenstephaner Steig 20, 85354 Freising, Germany; 2 Chair of Bioseparation Engineering, Technical University of Munich, TUM School of Engineering and Design, Boltzmannstr. 15, 85748 Garching, Germany; 3 Division of Medicinal Chemistry, Medical University of Graz, Otto-Loewi Research Center, Neue Stiftingtalstr. 6, 8010 Graz, Austria; 4 BioTechMed-Graz, Graz, Austria

**Keywords:** Yeast, Physiological state, Viability, Plasma membrane, Membrane fluidity, Nanoparticles

## Abstract

For industrial processes, a fast, precise, and reliable method of determining the physiological state of yeast cells, especially viability, is essential. However, an increasing number of processes use magnetic nanoparticles (MNPs) for yeast cell manipulation, but their impact on yeast cell viability and the assay itself is unclear. This study tested the viability of *Saccharomyces pastorianus ssp. carlsbergensis* and *Pichia pastoris* by comparing traditional colourimetric, high-throughput, and growth assays with membrane fluidity. Results showed that methylene blue staining is only reliable for *S. pastorianus* cells with good viability, being erroneous in low viability (*R*^2^ = 0.945; $\widehat{\sigma }$ = 5.78%). In comparison, the fluorescence microscopy–based assay of *S. pastorianus* demonstrated a coefficient of determination of *R*^2^ = 0.991 at $\alpha =0$ ($\widehat{\sigma }$ = 2.50%) and flow cytometric viability determination using carboxyfluorescein diacetate (CFDA), enabling high-throughput analysis of representative cell numbers; *R*^2^ = 0.972 ($\alpha =0$; $\widehat{\sigma }$ = 3.89%). Membrane fluidity resulted in a non-linear relationship with the viability of the yeast cells ($\alpha \ne 0$). We also determined similar results using *P. pastoris* yeast. In addition, we demonstrated that MNPs affected methylene blue staining by overestimating viability. The random forest model has been shown to be a precise method for classifying nanoparticles and yeast cells and viability differentiation in flow cytometry by using CFDA. Moreover, CFDA and membrane fluidity revealed precise results for both yeasts, also in the presence of nanoparticles, enabling fast and reliable determination of viability in many experiments using MNPs for yeast cell manipulation or separation.

**Supplementary Information:**

The online version contains supplementary material available at 10.1007/s00216-023-04676-w.

## Introduction

Microorganisms react to changing environments [1]. For instance, yeast cells used in industrial processes adapt to parallel stress factors, such as medium composition, pH value, temperature, and metabolites [2].

This adaption process is possible because the yeast cell is separated from its environment by the cell wall that fulfils various functions [3] and has a rigid structure. The cell wall protects the interior of the yeast cell from external influences, such as changes in temperature, osmotic pressure, or mechanical forces. However, the cell wall must obtain fluid characteristics that enable it to adapt to cell growth, division, and osmotic changes and thus maintain its physiological state [3]. The physiological state of a yeast cell depends on the single-cell age as well as its vitality and viability.

The parameter of single-cell age defines the number of reproduction processes that a yeast cell experiences [4], which varies with yeast strain [5, 6] and growth conditions [7, 8]. A number of studies have shown that single-cell age impacts stress resistance and growth velocity [9]. Yeast vitality indicates that a single cell is metabolically active. The final constituent factor of the physiological state is the yeast cell’s viability, indicated by the number of living cells within a population. Yeast cell viability is measured using several established assays, such as methylene blue staining [10] and esterase activity, to determine the metabolic activity or colony-forming units as a method for growth capacity. Table 1 in the supplementary information gives an overview of the advantages and limitations of cell viability test assays and their usability in representative cell numbers.

Learmonth and Gratton [11] focused on yeast cell viability in dependence on membrane fluidity. The study was performed using microscopy, which enabled qualitative, albeit not statistically significant, statements. The study findings have created an interconnection between viability and membrane fluidity, determined by calculating the generalised polarisation (GP), as indicated by a high GP value for low-viability cultures and vice versa. Most other studies of membrane fluidity examine changes in fluidity due to cell stress [12, 13]. However, these studies do not focus on yeast cell-plasma membranes and their fluidity or rigidity in the context of viability. Yeast cells respond to environmental changes, such as nutrient deficiency, metabolite accumulation, temperature fluctuations, or pH maintenance, by adapting the plasma membrane structures [14]. An acidic cell growth environment lowers the intracellular pH value (ICP) in the cytosol, which activates the ATPase [15]. This makes the membrane prone to environmental influences [11], such as temperature [16, 17] or the presence of ethanol [13, 18]. The matrix of these membranes is a bimolecular mixture of phospholipid molecules in which the phospholipids align the polar chains with the outer surfaces of the bilayer.

In contrast, the membrane’s interior consists of hydrophobic lipid chains [19]. This means that small hydrophobic molecules can easily diffuse through the two-dimensional fluid membranes, but it would be difficult or even impossible for larger ones to pass through. Charged larger molecules cannot penetrate the membrane.

Knowledge relating to membrane fluidity can only be obtained from the fluorescence properties of membrane-associated dyes [20]. Membrane fluidity can be evaluated using the fluorescent dye *laurdan* by parallel measurement of the emission intensities at 440 and 490 nm [11]. Changes in the membrane’s water content cause shifts in the emission spectrum of laurdan, which can be quantified by calculating the GP [21]. To do this calculation, the fluorophore is excited at 360 nm, and the fluorescence is recorded at 440 and 490 nm emission wavelengths. The lower the water content of the membrane, the further the maximum emission shifts from 480 to 435 nm, resulting in a higher GP value [22].

There is a growing interest in the use of magnetic nanoparticles (MNPs) for yeast cell lysis [23], cell separation, fractionation or immobilisation [24], biosorption material in the form of magnetic yeast [25-27], and biosensor material. They are all easy and inexpensive to synthesise, have a high surface-to-volume ratio, and are biocompatible [28]. Recently, Loira et al. [29] reviewed the application of nanomaterials in the wine-making process. Among many applications, they state that MNPs are used in the protein clarification of white wine [29]. Moreover, the magnetic removal of yeast cells is an effective and cost-efficient way of separating cells during fermentation. Berovic et al. [30] developed a separation method consisting of a column filled with steel wool which is flown through by the feed to separate magnetically labelled yeast cells from sparkling wine without affecting the cell metabolism.

Some studies have indicated a toxic effect of nanoparticles on mammalian cells by the induction of ROS production and oxidative stress [31, 32]. The majority of studies evaluated the impact of silver [33], zinc [33], and copper [34] nanoparticles on yeast cells. The effect of iron oxide particles, confirmed as biocompatible, on yeast cells has not yet been fully described. Fraga-García et al. [35] published findings regarding the impact of bare, uncoated iron oxide magnetic NP (BIONs) on the growth of yeast cells, observing no negative impact. In ethanol biosynthesis, due to magnetised *Saccharomyces cerevisiae*, Firoozi et al. [36] evaluated by way of growth analysis that L-lysine coated MNPs do not affect the yeast cell viability up to 250 µg/mL. These results are confirmed by Tagizadeh et al. [37] who could observe no adverse effect of BIONs on the growth and viability of *P. pastoris*. Bare iron oxide nanoparticles have been given GRAS status (generally recognised as safe) by the US Food and Drug Administration (FDA) and have accordingly been approved for in vivo applications [38]. For example, they are suitable for medical, biotechnological, and food technology applications (e.g. DNA and protein purification and for separating microbiological cells [35, 39]). However, it appears that some studies observed a negative impact of BIONs on viability, apoptosis, and intracellular ROS generation in yeast cells [40, 41]. Paunovic et al. [40] emphasise the lack of high-quality studies, as well as the impact of morphology, size, and surface area, which are highly dependent on the medium used. The cytocompatibility of the BIONs can be increased by embedding them in biocompatible shells such as bovine serum albumin (BSA) [42] or dextran [43]. Nevertheless, the application of BIONs with or without functionalisation is of interest in the future separation of yeast cells using specific protein-binding proteins [8, 44].

To sum up, several methods are currently in use to determine cell viability in the presence of MNPs. Still, no studies have focused on the impact of BIONs on the assay itself, resulting in false positives or false negatives. Therefore, it is essential to compare established approaches under the same conditions.

This study analysed a new approach for yeast viability determination, which consists of measuring the membrane fluidity explained by the term of generalised polarisation of yeast cells using laurdan, a solvatochromic fluorescent dye that changes its colour in dependency on the water content of the plasma membrane. Learmonth and Gratton [11] show that low viability relates to high fluidity; however, no comparison was made with established assays and in stress conditions (e.g., alcohol concentrations). In comparison with further publications, e.g. Kwolek-Mirek and Zadrag-Tecza [45], we determined the precision of yeast viability assays using two industrial-relevant organism samples of defined viability rather than a single sample. As stated by van Zandycke et al. [46], the precision of methylene blue is disputed, so it is necessary to compare the precision according to the yeast cells’ viability. In addition to comparing membrane fluidity with established assays, we focused on the impact of nanomaterials, such as magnetic particles, on both assay performance and yeast viability, measured using these comparison methods.

## Materials and methods

### Strain and strain maintenance

This study used the bottom-fermenting yeast *Saccharomyces pastorianus* var. *carlsbergensis* TUM 34/70 and the *Komagataella phaffii* (*Pichia pastoris*) strain BSY BG11 (Bisy GmbH, Hofstaetten). The organisms were grown on yeast extract peptone dextrose (YPD) agar plates (10 g L^−1^ Bacto yeast extract, 20 g L^−1^ Bacto peptone, 20 g L^−1^ glucose, and 10 g L^−1^ agarose). A YPD medium was inoculated to 1 million cells mL^−1^ cell concentrations for viability determination and incubated for 20 h under agitation at 120 rpm and 18 °C, resulting in a stationary phase culture.

### Viability determination assays

The yeast sample in all viability assays was divided into two fractions: one with viable yeast and the other with dead yeast. Viable yeast was heated for 10 min at 100 ℃; after which, the cell concentrations were determined and adjusted to the same optical densities, and distinct dilutions of viability (0%, 12.5%, 25%, 37.5%, 50%, 62.5%, 75%, 87.5%, 100%) were made using the two yeast viability fractions. All viability determinations were performed with three independent diluted samples.

1. Methylene blue is a compound used for staining microscopic organs to be examined under a microscope [10]. It has also been used to approximate the number of viable cells in a yeast sample [47]. Accordingly, the yeast cells were diluted. A 500 µL yeast dilution was added to 500 µL of methylene blue (0.01% (w/v) in 2% (w/w) sodium citrate), and the solution was incubated for 5 min at room temperature. After incubation, the yeast cells were analysed microscopically by counting at least 150 cells per viability mixture. This method of determining cell viability using methylene blue is less accurate than other methods since, according to the literature. the methylene blue assay overestimates living cell content [46, 48].
2. Methylene violet is an alternative method for determining yeast viability. The yeast cell suspension was prepared in a similar way to methylene blue staining. A methylene violet staining solution was prepared by dissolving methylene violet (0.1% (w/v) in distilled water. The resulting solution was diluted tenfold in a glycine buffer (pH 10.6), and 0.5 mL methylene violet solution was mixed with 0.5 mL of yeast suspension.
3. Mg-ANS, the magnesium salt of 1-anilino-8-naphtalene sulphonic acid, is a protein-binding fluorescent dye and was described by McCaig [49]. Yeast cell mixtures were mixed with an equal volume of a 0.3% solution of Mg-ANS and incubated for 5 min at room temperature. Non-viable cells were stained green due to stained proteins; they were excited at 365 nm and counted by the emission at 445 nm. Viability was calculated by counting the fluorescent cells and then determining brightfield’s total cell number with at least 150 cells per sample.
4. Counting colony-forming units is one of the traditional methods of determining viability [50, 51]. A defined volume of yeast suspension was spread on YPD plates and incubated at 30 °C for 2 days, in accordance with Wang et al. [51]. The colonies were counted manually, and their viability was determined in relation to the total cell number.
5. The tadpole assay is a simplified test to determine the number of colony-forming units [50]. To do this, 220 µL of each mixture was transferred to the first 96-well plates. The volume was serially diluted by one to 10 (20 µL culture in 180 µL medium), ten times. The well plate was then incubated at 25 ℃ for 2 days and colonies were counted.
6. Finally, carboxyfluorescein diacetate (CFDA) was used to measure the cells’ metabolic activity. CFDA passes through the yeast cell membranes and becomes metabolised to the fluorescent product of carboxyfluorescein. The cell is stained in the case of viable yeast cells, but non-viable yeast cells are unstained. The method is based on the work of Weigert et al. [52]. The sample was prepared on ice. The supernatant of a 2 mL yeast suspension (*OD*_600_ = 1) was decarded after centrifugation for 3 min at 7000 g. The cells were washed three times in an ICP buffer (McIlvaine buffer (pH 3) with an additional 110 mM of sodium chloride, 5 mM potassium chloride, and 1 mM magnesium chloride) and resuspended in 2 mL ICP buffer. Fifty microliters of the suspension was made up to 2 mL using the ICP buffer. After adding 2 μL of carboxyfluorescein diacetate solution (10 mM in DMSO), the mixture was incubated for 10 min at 30 ℃ and protected from light. The subsequent measurement was conducted by flow cytometer, as described later. The detectable fluorescence differs between non-viable and viable cells.

### Membrane fluidity measurement

Membrane fluidity is the parameter that describes how yeast cells react to environmental changes. Furthermore, ergosterol is required for a defined regulation of membrane fluidity. To measure membrane fluidity, the yeast cell suspension was adjusted to an optical density at 600 nm of 0.4. Five microliters of laurdan (15.9 mg in 9 mL ethanol and 41 mL DMF) were added to 1 mL of this yeast suspension, resulting in a final laurdan concentration of 5 µM. Samples were then incubated in the dark under the same growth conditions as the yeast suspension. After incubation, the fluorescence intensity of a sample with a volume of 200 µL was measured at an excitation of 360 nm and emissions of 440 and 490 nm in a microtiter plate, using a Cytation5 multi-plate reader (Biotek Instruments) at 30 °C. Using these two fluorescence detectors, the generalised polarisation was calculated according to [53]:

$$GP= \frac{{I}_{440}-{I}_{490}}{{I}_{440}+{I}_{490}}$$

A high GP value is related to a low membrane fluidity, corresponding to a more gel-phase liquid-ordered membrane. Low GP values correspond to a liquid-disordered membrane structure and a more fluid membrane [11].

### Fluorescence-coupled flow cytometry

Flow cytometry measurements of fluorescence intensities of unstained and stained yeast cells were conducted using a Cytoflex cytometer (Beckman Coulter) with an argon ion laser (15 mW laser power with excitation wavelength 488 nm). Carboxyfluorescein fluorescence was detected on the FL1 channel (525 nm), and FL2 channel (585 nm) with at least 20,000 cells in each analysis (sample flow: max. 150 events s^−1^; gain: 100). The FSC and SSC detectors (gain: 500) were measured as signals to differentiate between yeast cells and particles. Each yeast suspension was analysed in independent triplicate samples.

### Membrane fluidity reaction to alcohol stress

To analyse the impact of alcohol stress (ethanol for *S. pastorianus* and methanol for *P. pastoris*) on membrane fluidity, metabolically active cells of the exponential growth phase were exposed to increasing alcohol concentrations (0 to 6% in 1% steps, diluted in YPD medium), which led to the loss of yeast viability. The impact of this stress was measured by comparing the change in membrane fluidity (determined by laurdan) with the loss of cell viability obtained by counting CFU after 1 h, 3 h, and 5 h of incubation.

### Determining the viability of stressed brewers’ yeast

To test the reliability of the assays with exponential cell growth in an industrial environment, membrane fluidity, CFU, and CFDA assays were performed on yeast cells grown anaerobically in wort at 12 °C for 1 day, 7 days, and 14 days. To do this, 10 mL of wort was inoculated to a cell density of 10 million cells mL^−1^ in a 50-mL flask and sealed with a fermentation tube to create an anaerobic environment. The flask was shaken in an incubator at 120 rpm. The viabilities of the samples after 1, 7, and 14 days were compared to the initial viability, as determined by all three assays.

### Synthesis of magnetic nanoparticles

Bare iron oxide nanoparticles (BIONs) were synthesised by co-precipitation of Fe^2+^/Fe^3+^ ions, as described by Turrina et al. [54]. First, a solution of 28.9 g sodium hydroxide (723 mmol, 10.3 equivalents (eq.) Carl Roth GmbH + Co. KG) in 400 mL of degassed water was mixed with a solution of 34.6 g FeCl_3_·6H_2_O (128 mmol, 1.82 eq., Sigma-Aldrich Merck KGaA) and 14.0 g FeCl_2_·4H_2_O (70.4 mmol, 1.0 eq., Sigma-Aldrich Merck KGaA) in 160 mL of degassed water under nitrogen atmosphere and agitated at 27 ℃. The reaction continued for 30 min. Then, the black precipitate was washed in deionised water by magnetic decantation with a neodymium iron boron magnet in a glass bottle until the conductivity was below 200 µS cm^−1^. The BIONs were stored under nitrogen at 4 ℃. Turrina et al. [54] subjected the particles to a thorough characterisation and showed that the BIONs displayed superparamagnetic behaviour without significant remanence. Transmission electron microscopy (TEM) was performed with a JEOL JEM 1400 Plus microscope, and the recorded images were evaluated using ImageJ software. Diluted nanoparticle suspensions were precipitated on carbon-coated copper grids before the TEM measurements. The average diameter was determined from more than 100 evaluated particles.

The hydrodynamic diameter was measured by dynamic light scattering (DLS) with ZetaSizer XS (Malvern Panalytical GmbH), and zeta potential. Both measurements were performed at 25 ℃ in 1 mL of a 1 g L^−1^ or 0.1 g L^−1^ solution, respectively, conducted in three measurement cycles in a 20 mM 3-(N-morpholino)propanesulfonic acid (MOPS) buffer pH 8.

### Impact of nanoparticles on yeast cells’ viability and viability assay performance

To test whether nanoparticles impact the performance of the aforementioned viability assays, yeast cells were mixed with bare magnetic nanoparticles. The magnetic nanoparticle concentrations were diluted using 20 mM MOPS (pH 8) in two testing concentrations: 0.1 and 1 g L^−1^. The dilution was mixed with yeast cells at different viabilities, resulting in a final optical density of 1 at 600 nm.

### Data analysis

Classification of viable and non-viable cells using CFDA is performed using the fluorescence quotient of 525 nm and 585 nm detector signals. Particles with a 525 and 585 nm fluorescence quotient above 2 and fluorescence intensities at 525 nm above 20,000 for *S. pastorianus* and 5000 for *P. pastoris* were viable, while particles below that were non-viable.

Nanoparticles and yeast cells are classified using a random forest model.

For this purpose, fluorescence data of 20,000 nanoparticles were designated target “0” and yeast cells with were designated target “1.” Data was added to the nanoparticle dataset, and the whole randomised dataset was divided into training data (0.75) and test data (0.25). The training data was used for random forest model training using FSC and SSC channels. The test data for model evaluation resulted in an *R*^2^ of 0.91.

## Results and discussion

To define a precise method of determining yeast viability, the following approaches were compared: (I) methylene blue/methylene violet, (II) Mg-ANS (both microscopic approaches), (III) colony-forming units (CFU) and (IV) tadpole assay (both growth assay), (V) carboxyfluorescein diacetate (CFDA) as a cytometric assay, and (VI) membrane in a fluidity fluorescence approach.

All assays were calibrated using *S. pastorianus* and *Pichia pastoris* (*Komagataella phaffii*) yeast cells with defined viability by mixing high-viability yeast cells and dead cell cultures. Furthermore, yeasts were exposed to alcohol in different concentrations, and membrane fluidity values were compared to CFU values.

To determine whether nanoparticles impact the yeast cells’ viability, we also tested 100% viability and 62.5% viability yeast in two different nanoparticles concentrations after 1 h, 3 h, 6 h, and 24 h using CFDA and membrane fluidity. The viability assays were also tested for their reliability using BIONs, as they tend to influence optical analytics due to their high absorbance level.

### Generalised polarisation measurement for determining membrane fluidity

**Fig. 1:**
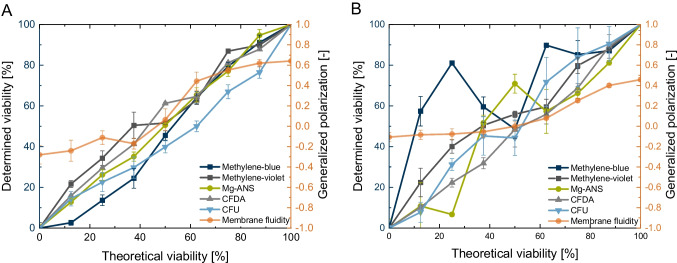
Comparison of viability assays: (i) microscopic colourimetric assay conducted by staining yeast cells with methylene blue (117–209 counted cells per sample), methylene violet (109–219 counted cells per sample), and Mg-ANS (112–209 counted cells per sample); (ii): cytometric assay conducted by staining yeast cells with CFDA and subsequently distinguishing according to fluorescent and non-fluorescent yeast (20,000 cells per sample); (iii): growth assay conducted by counting cfu (stock solution: 8781 cells µL^−1^); (iv): fluorescent-spectroscopic assay conducted by measuring membrane fluidity (8.9 × 10^6^ cells mL.^−1^). **A** *S. pastorianus* ssp. *carlsbergensis* cells; **B** *P. pastoris* cells. *N* = 3

The plasma membrane fluidity of yeast cells is determined with generalised polarisation (GP), as reported in the literature [22, 55]. Learmonth and Gratton’s [11] previous results showed an interconnection between membrane fluidity and the viability of yeast cells. They showed that viable yeast cells were determined with a high GP value, as measured by the quantification of microscopic fluorescence intensity. We used yeast cell mixtures of defined viability to determine the variance between actual viability and generalised polarisation. Our results indicate the same trends as those presented by Learmonth and Gratton [11], and we also confirmed a higher GP value, corresponding to a higher yeast cell viability (Fig. 1 A). Conversely, a low viability would correlate with a negative GP value. Specifically, a viability of 100% matches a GP value of 0.64, and a viability of 50% is related to a GP value of 0.064 for *S. pastorianus*. It is important to note that the membrane fluidity is calculated as a sum term of all cells.

We also demonstrated that a viability above 62.5% has a detectable signal difference. Below 50% viability, there is a low linear correlation. This regression indicates a non-linear logistic relationship between yeast cell viability and membrane fluidity, with a coefficient of determination of 0.994. Therefore, in the case of good viability, membrane fluidity can be regulated by rearranging the membrane composition. The number of dead cells affects the fluidity of the overall population. If the viability is below 50%, the weight of dead cells is higher, and, in turn, the error is also higher. Single-cell analysis, such as flow cytometry, is a more suitable approach than this assay. Flow cytometry allows cell fragments to be removed from the data analysis, enabling more precise determination. In a low-viability concentration, a higher error rate was measured due to cell fragments or cell walls caused by the heating step of the 0% viability sample. However, a disadvantage of this approach is that it involves a sum term, which is affected by the manual error and cell number inhomogeneity. Also, many factors affect membrane fluidity, such as ethanol concentration [13, 18] and heat stress [56]. Therefore, a new calibration curve needs to be performed for each condition.

Next, we apply the membrane fluidity approach to *P. pastoris* cells (Fig. 1 B). Results comparable to *S. pastorianus* were detected for this strain. A linear interconnection is obtained above a viability of 62.5%. In contrast, below 50% viability, the assay is erroneous, as indicated by high error bars. Overall, the measured membrane fluidity values of *Pichia pastoris* are lower than for *S. pastorianus*, indicating a lower ergosterol content in the plasma membrane. This observation agrees with the results obtained by Grillitsch et al. [57], who determined an eight-times lower ergosterol concentration in *P. pastoris* cells compared to *S. cerevisiae* cells.

### Comparison with established viability determination assays

To evaluate a precise method of determining yeast cell viability, we compared the results of membrane fluidity with established microscopic and growth methods. Figure 1 presents a comparison of all five assays, of which methylene blue staining is the most conventional. Viable yeast cells have enzymes that remove the colour in methylene blue; however, this is not the case with dead cells [58]. Therefore, when yeast cells are placed in a solution with dye, the dead cells are stained blue while the living cells remain unstained. The methylene blue assay displays a correlation of 0.945 between the theoretical and experimental viability using *S. pastorianus* cells (Fig. 1 A). Methylene violet and methylene blue displayed a Pearson *R*^2^ of 0.966. However, for *P. pastoris* cells, as shown in Fig. 1 B, the correlation is much poorer, at *R*^2^ = 0.207, with a standard regression error $\widehat{\sigma }$ of 20.27%.

In contrast, methylene violet is much more precise for *P. pastoris* cells, indicating an *R*^2^ of 0.93 between theoretical and determining viability. The generalised polarisation has an interconnection towards viability resulting from significant differences when the viability is above 50%. Mg-ANS staining shows a more precise correlation in viability over the full range of 0–100% (*R*^2^ = 0.991 for *S. pastorianus* ($\widehat{\sigma }$ = 2.50%) and *R*^2^ = 0.874 for *P. pastoris* ($\widehat{\sigma }$ = 12.62%)). Thus, the Mg-ANS assay is preferred with microscopic assays because of the coefficient of determination and low standard error of the regression. On the other hand, this assay is impractical in laboratories that do not possess the required fluorescence equipment. In addition, manual counting is time-consuming due to the switch between fluorescence and transmission light.

In comparison, flow cytometric determination of viability using CFDA by distinguishing between fluorescent and non-fluorescent particles was performed by dividing the fluorescence intensity at 525 nm and 585 nm. Carboxyfluorescein diacetate is metabolised in living yeast cells because of esterase activity. Dead cells are non-fluorescent due to the lack of metabolic activity. Viability determination using CFDA resulted in a correlation of 0.972 for *S. pastorianus* ($\widehat{\sigma }$ = 3.88%) and 0.984 for *P. pastoris* ($\widehat{\sigma }$ = 3.21). In contrast to the earlier assays, many cells were analysed in the CFDA assay, which is, therefore, statistically significant. According to a cell number of unlimited, as occurs at a yeast cell concentration above 1 × 10^6^ cells mL^−1^ and an expected error rate of 5%, a cell count of 385 is necessary for a significant population expression [59]. This cell number does not occur in most microscopic methods in the literature. None of these assays was confirmed by calibrating with different *Pichia pastoris* viabilities (in the literature, for *Pichia pastoris* methylene blue assays [60, 61], CFU [62], or more recently by the use of commercial staining assays determining the esterase activity [63, 64]). In the viability determination of *Clostridium* spp. using CFDA and flow cytometry, a high coefficient of determination was examined (0.990 ± 0.006 and 0.996 ± 0.003) [65]. Boyd et al. [66] examined similar results by comparing methylene blue staining and flow cytometry measurements. These authors determined an *R*^2^ of 0.87 between flow cytometric- and methylene blue–determined viability. In addition, they concluded that methylene blue overestimated viability. Mg-ANS also correlates with results obtained by slide cultures [49]. We were able to confirm these results by correlating theoretical and experimental viability.

A tadpole assay showed a lack of differentiation between the colonies over the whole range, leading to unprecise results. As stated in the research by Welch et al. [20], the necessary incubation period is 2–3 days at 30 ℃. In this study, an incubation time of 2 days at 20 ℃ resulted in an uncountable number of colonies in the liquid medium. Optimisation of this assay is therefore necessary. For this purpose, conventional CFU was used to compare the assays, resulting in an *R*^2^ of 0.937 for *S. pastorianus* ($\widehat{\sigma }$ = 5.17%) and 0.960 for *P. pastoris* ($\widehat{\sigma }$ = 5.912).

All methods were conducted in accordance with the published experiment protocols. A Pearson correlation of all assays together indicated a positive correlation of > 0.941 for *Saccharomyces* yeast and > 0.940 for *Pichia pastoris*, except methylene blue (0.759). In the next step, the reliability of the assays with industrial samples was investigated. Three samples were taken from *Saccharomyces* fermentation at different time points, and the assays of methylene blue, Mg-ANS, CFDA, and cfu were compared with each other. The results in Figure S1 in the SI confirm our previous findings from the assay comparison with defined viability amounts. From Figure S1, we can conclude that colony-forming units and CFDA staining indicate the same amount of viability and are independent of the fermentation compounds.

Overall, the CFDA assay shows high precision for both yeast strains over the entire viability range. Furthermore, membrane fluidity is a reliable assay for both yeast strains in the case of high viability.

### Impact of alcohol stress on membrane fluidity and viability

Based on the knowledge that alcohol impacts membrane fluidity and, in turn, the calibration curve, this effect was determined by inoculation of *S. pastorianus* yeast cells in ethanol and *P. pastoris* in methanol.

**Fig. 2:**
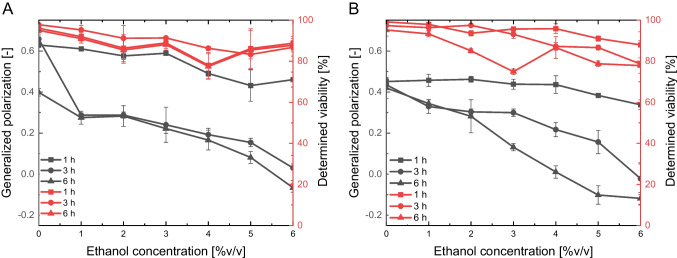
Comparing the impact of alcohol stress on yeast viability, as determined by membrane fluidity (black) and colony-forming units (red); **A** *S. pastorianus* ssp. *carlsbergensis*; **B** *Pichia pastoris*; cfu: 155–209 counted cells per sample; membrane fluidity: 10 × 10^6^ cells mL.^−1^; *N* = 3

We observed a change in membrane fluidity according to time and alcohol concentration (Fig. 2). This interconnection has been published in many previous studies, which indicated that alcohol results in fluidisation of the membrane [12, 18, 67] but can also be seen as an indicator of ethanol tolerance [12]. Our study has shown that increasing ethanol concentrations increase membrane fluidity, evidenced by a decrease in the generalised polarisation. Furthermore, a process of adaptation can be seen in the membrane fluidity, demonstrated by a further reduction in generalised polarisation over time caused by the higher ethanol concentration. A correlation of the effect by the Pearson correlation coefficient between ethanol concentration and cfu produced values of 0.44 after 1 h, 0.70 after 3 h, and 0.07 after 5 h for *S. pastorianus*. For *P. pastoris*, a value of 0.89 was calculated after 1 h, 0.96 after 3 h, and 0.64 after 5 h. Focusing on these calculated values, a slight interconnection can be determined between alcohol content and viability. We thus concluded that the increase in fluidity and, in turn, the decrease in the generalised polarisation, was due to the alcohol concentration. Therefore, a correlation between viability and membrane fluidity could be determined by performing a multidimensional calibration between the parameter of alcohol and yeast viability, whereas knowing the alcohol concentration enables the viability to be calculated.

### Characterisation of the bare iron oxide nanoparticles (BIONs)

**Fig. 3:**
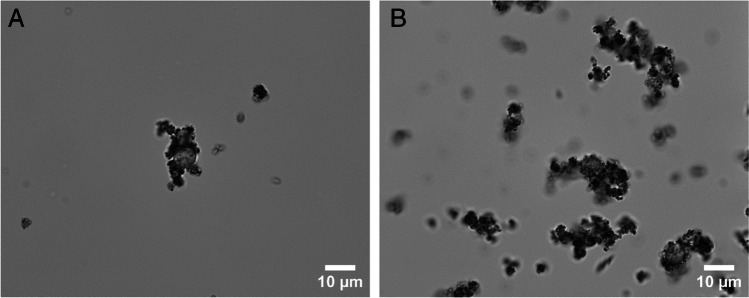
Light microscopic images of *S. pastorianus* (*OD* = 1) mixed with a nanoparticle concentration of 0.1 g L^−1^ (**A**) and 1 g L^−1^ (**B**) in 20 mM MOPS pH 7.3 after 5 min incubation time

Before investigating the influence of the BIONs on the viability of the yeast cells, they are analysed to determine their properties. The results of this characterisation are given in the supplementary information section of Figure S2. Figure 3 A and B show light microscopic images of a mixture consisting of 0.1 g L^−1^ (A) and 1 g L^−1^ BIONs (B) with *S. pastorianus*. The nanoparticles agglomerate, adsorbing unspecific onto the cell surfaces, as previously noted in the literature [30].

### Performance of yeast viability determination assays in the presence of iron oxide nanoparticles

To compare the assay performance in the presence of magnetic nanoparticles, particle dilutions were mixed with *S. pastorianus* yeast cells, and the viability was determined.

**Fig. 4:**
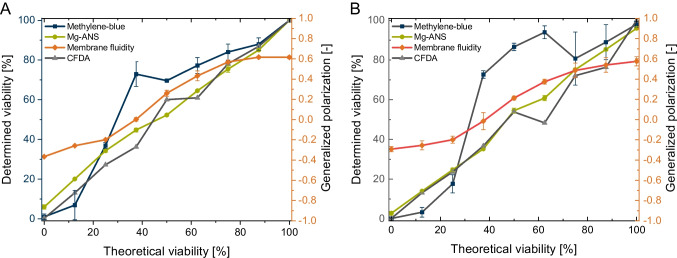
Comparison of four viability determination assays with *S. pastorianus* cells; **A** immediately after nanomaterial contact; **B** after 1 h of nanomaterial contact; nanoparticle concentration = 0.1 g L^−1^; *N* = 3; methylene blue: 30–643 counted cells per sample; Mg-ANS: 101–342 counted cells per sample; membrane fluidity: 8.9 × 10^6^ cells mL^−1^; CFDA: 15,000 particles per sample

Figure 4 compares the results for a particle concentration of 0.1 g L^−1^. The methylene blue and methylene violet staining assay (methylene violet data not shown) is disturbed to a high degree, representing high viability. This effect can be explained by the adsorption effect of nanoparticles, as described by Talbot et al. [68], in which the authors reported an adsorptive effect of organic dyes on nanoparticles. As a result of this effect, methylene blue is complexed, and the dye concentration in the assay is reduced, resulting in lower dyeing and higher determining viability.

**Fig. 5:**
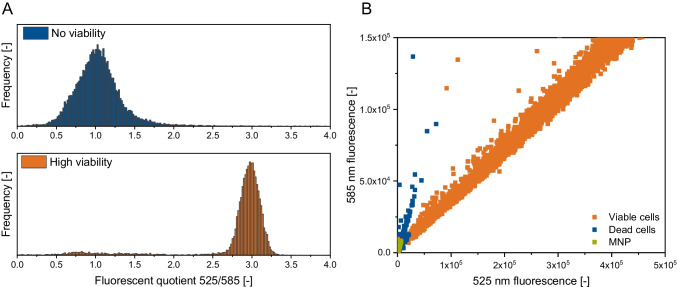
**A** Comparison of the fluorescence quotient of high-viability yeast cells and dead yeast cells, calculated by dividing the fluorescence intensity at 525 nm by 585 nm; **B** Differentiation of magnetic nanoparticles (MNP), high- and low-viability yeast cells, by the fluorescence intensities at 525 and 585 nm; *N* = 20,000

We can report that fluorescence-based assays, such as Mg-ANS microscopy or membrane fluidity measurement, are not disturbed by nanoparticles. Nanoparticles have the advantage of having a low and constant fluorescence, as shown by Shi et al. [69]. Membrane fluidity measurement yields an exact determination of the yeast cells’ viability. In addition, flow cytometry measurement using CFDA is unaffected by the presence of nanoparticles. However, thanks to the single-particle analysis, nanoparticles can be differentiated from high- and low-viability yeast cells based on the fluorescence intensity, as shown in Fig. 5.

Figure 5 compares a high-viability fluorescence quotient and a non-viable yeast. The median quotient of high-viability yeast is around 2.9. Only a small number of dead cells are present, as demonstrated by the first peak at the quotient of 0.85.

Furthermore, by focusing on the fluorescence intensities at 525 and 585 nm, differences between viable cells, dead cells, and magnetic nanoparticles can be seen (Fig. 5 B). Nanoparticles have a low intensity and can be differentiated by these properties.

In addition, measurements indicate that a particle concentration of 1 g L^−1^ is high for microscopic evaluation. Particles agglomerate to a certain degree, disabling the differentiation between yeast cells and particle agglomerates, demonstrated in Fig. 3). In the case of one dead yeast sample (viability of 0%), no blue cells were visible using methylene blue staining. Pospiskova et al. [70] created a magnetic response in yeast cells by mixing yeast cells with microwave-synthesised iron oxide microparticles and observing agglomerates [70]. Tálos et al. [62] reported that yeast cells have a negative surface charge, and the zeta potential of the particles is also negative. However, a local electrostatic interaction with positively charged yeast cell wall domains is reported by Bos et al. [71]. A similar surface charge explains the adsorptive effect of the cation methylene blue on nanoparticles [72].

No significant impact between the methods could be determined when comparing all three assays—Mg-ANS, CFDA, and membrane fluidity. Although Mg-ANS has the advantage of determining precise microscopic viability, it focuses on a counting technique. The disadvantage of such techniques is that it involves manually counting a small number of cells, with deviations occurring between different persons. However, CFDA combined with flow cytometry is precise, until it counts a defined number of cells in less time, enabling a single-cell analysis.

Based on the results from “Comparison with established viability determination assays,” we did not compare all assays with *P. pastoris* cells. Furthermore, we analysed the impact of nanoparticles on *P. pastoris* by flow cytometry and membrane fluidity, using a known viability of 50% and 100%. The results are shown in Table S2 in the supplementary information.

### Impact of nanomaterials on the viability of yeast cells over time

In established yeast separation processes, yeast cells were buffered in PBS or buffers with low molarity, as the medium impacts the viscosity. Therefore, this environment changes particle behaviour and effectivity, especially by working with nanoparticles. Furthermore, the separation process takes time, and the impact of the mixture of nanoparticles and time on yeast cells’ physiological state has to be determined.

**Fig. 6:**
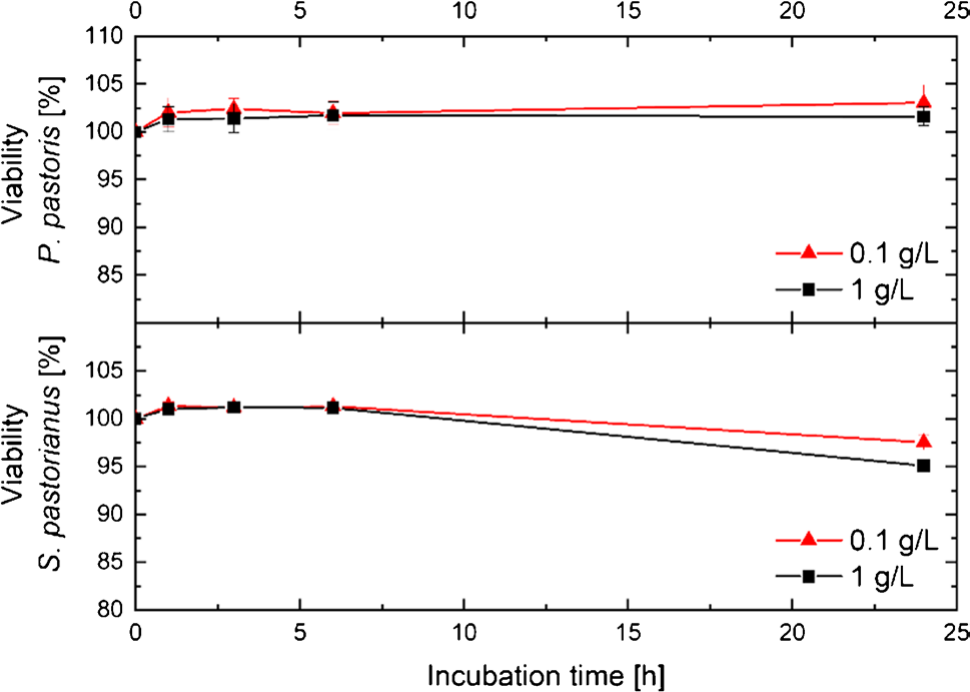
Yeast viability trend (determined by flow cytometry using CFDA) in the presence of 0.1 and 1 g/L nanoparticle concentrations over 24 h. Yeast cell viability was standardised to the viability of yeast cells without nanoparticles; top: *P. pastoris* yeast; bottom; *S. pastorianus* yeast; *N* = 20,000 particles including nanoparticles; after differentiation: 0.1 g/L: minimum 14,500 cells; 1 g/L: 3500 cells

To measure the impact of nanoparticles on yeast cell viability over time, the viability of *S. pastorianus* and *P. pastoris* was again determined by CFDA, after 1 h, 3 h, 6 h, and 12 h. On comparing the effect of nanoparticles on *S. pastorianus* and *P. pastoris* yeast cells for more than 1 h, no impact on yeast viability could be determined, as seen in Fig. 6. By focusing on a yeast sample with a theoretical viability of 100%, Fig. 6 shows the changes over 24 h, determined by CFDA staining.

No significant impact of nanoparticles on yeast cells could be determined within a timeframe of 6 h for both yeast strains, *S. pastorianus* and *P. pastoris*. After 24 h, a slight decrease in *S. pastorianus* viability could be detected. In contrast, *P. pastoris* cells were highly viable after 24 h of incubation with both nanoparticle concentrations. Peng et al. [73] determined the effect of magnetic iron oxide particles on yeast cells, resulting in the involvement of particles in mitochondrial dysfunction. They concluded that nanoparticles have a toxic effect on the organisms but that this effect greatly depends on the particle concentration [73]. Comparable results were determined by Firoozi et al. [36], who immobilised iron oxide nanoparticles on yeast cells.

In contrast, we showed that nanoparticles have a slightly negative effect on *S. pastorianus* yeast cells by reducing their viability by 5% in a 1 g L^−1^ concentration, but they do not affect *P. pastoris*. In biomedical applications, iron oxide nanoparticles are used with a customised coating, which enables specific functionalisation, enhances their biocompatibility, and controls their behaviour [74] by reducing their toxic effect on cells [75]. We demonstrated the worst-case scenario by testing the impact of BIONs without functionalisation on yeast cells for use in magnetophoretic processes (e.g. concentration, separation). These results provide the first experimental data on the nanoparticle impact on *Pichia pastoris* cells and are the basis for further experimental setup using the industrial-relevant yeast cells. For example, using this data from our study, *Pichia pastoris* cells can be coupled with nanoparticles for magnetic manipulation of these cells. This yeast cell manipulation approach can help separate yeast cells by surface characteristics for basic research on cell-dependent recombinant protein synthesis or metabolite formation.

## Conclusion

This study compares the method of membrane fluidity or fluorescence-coupled flow cytometry measurement as an alternative for determining yeast cell viability. The advantage of membrane fluidity is its easy handling and the representative number of cells analysed. We showed that membrane fluidity measurements follow a non-linear relationship, depending on the environmental composition (e.g. alcohol). In the case of low viability, a higher error rate was measured, making this approach unusable for unknown viabilities and environments. In contrast, flow cytometry precisely analyses a representative number of cells, independent of viability and yeast strain. A disadvantage of the membrane fluidity assay is the need to calibrate each yeast strain. Flow cytometry analyses particles by their fluorescence and scatter behaviour, enabling a quantitative differentiation of different particles.

We also investigated nanoparticles' lack of influence on yeast cell viability. We showed that nanoparticles reduce the amount of methylene blue, resulting in overestimated viability.

We also showed that nanoparticles do not affect yeast cell viability over 6 h. After 24 h, a slight reduction in the viability of *S. pastorianus* and *P. pastoris* could be determined. In addition, membrane fluidity measurement in generalised polarisation is a stable and precise method with yeast cell viability above 50%. We successfully demonstrated that CFDA and membrane fluidity measurements to determine yeast viability are relatively reliable techniques in the presence of nanoparticles.

These results highlight for the first time the potential of using magnetic nanoparticles in yeast cell fermentation without affecting the cells’ viability, thus enabling further applications that combine the metabolic activity of yeast cells with the magnetic properties of nanoparticles [76],﻿ e.g. magnetic cell separation for yeast repitching. They can also be used for magnetic separation of segregated proteins or cells without influencing cell viability. The application of a yeast separation process could enable the specific analysis of single yeast cells or the use of cell fractions for further fermentations.

## Supplementary Information

Below is the link to the electronic supplementary material.

Supplementary file1 (DOCX 215 KB)
