# Supplementary material for: Quantification methods of determining brewer’s and pharmaceutical yeast cell viability: accuracy and impact of nanoparticles
Source: Anal Bioanal Chem. 2023 Apr 21;415(16):3201–13. doi: 10.1007/s00216-023-04676-w (PMC10287788; doi:10.1007/s00216-023-04676-w)
Supplement: Supplementary file 1 — Supplementary file1 (DOCX 215 KB) [file 216_2023_4676_MOESM1_ESM.docx]

**Supplementary information**

Quantification methods of determining brewer’s and pharmaceutical yeast cell viability: Accuracy and impact of nanoparticles

*Eigenfeld, M.^1^; Wittmann, L. ^2^, Kerpes, R.^1^; Schwaminger, S.^2,3,4^, Becker, T.^1^*

^1^Technical University of Munich, TUM School of Life Science, Chair of Brewing and Beverage Technology, Weihenstephaner Steig 20, 85354 Freising, Germany

^2^Technical University of Munich, TUM School of Engineering and Design, Chair of Bioseparation Engineering, Boltzmannstr. 15, 85748 Garching, Germany

^3^Medical University of Graz, Otto-Loewi Research Center, Division of Medicinal Chemistry, Neue Stiftingtalstr. 6, 8010 Graz, Austria

^4^ BioTechMed-Graz, Austria

Corresponding author: Roland Kerpes, E-mail: roland.kerpes@tum.de

**Introduction:**

**Table S1:** Comparison of established viability assays according to measurement principle, advantage and limitation

| Assay approach | Principle | Advantage | Limitation |
| --- | --- | --- | --- |
| Methylene blue (MB) | **Microscopic approach**  Living cells are unstained and colourless, whereas dead cells are blue [1].  Two theories about the mechanism:  (i) methylene blue incorporates dead cells due to a defect membrane [2].  (ii) methylene blue is metabolised in dead cells to leuco-methylene blue [3]. | - Easy to handle - Only a microscope is necessary - Fast staining | - Cell counting is time-consuming - Non-representative cell numbers - Cell number depends on the person counting cells - Only applicable in defined cell density ranges (150 to 300 cells per counting chamber) - Unprecise at lower viabilities [4] - For comparison between measurements, a defined incubation time is recommended |
| Methylene violet (MV) | **Microscopic approach** | - Higher accuracy compared to methylene blue [1] - Fast staining and easier differentiation between living and dead cells compared to MB - Does not require experienced knowledge | - Only an alkaline solution can be recommended [5] - Non-representative cell numbers - Unprecise with yeast cells of high repitching number [6] - Only applicable in defined cell density ranges (150 to 300 cells per counting chamber) |
| 1-anilino-8-naphtalene sulphonic acid magnesium salt (Mg-ANS) | **Microscopic approach**  Living cells are unstained, whereas dead cells are fluorescent blue [7] due to a lack of membrane permeability. | - Precise viability determination - Minor damages are visible due to fluorescence - Independent of incubation time | - Same disadvantages as MB - Fluorescence equipment necessary - No second dye disables differentiation between subpopulations - Non-representative cell numbers |
| Carboxyfluorescein-Diacetate (CFDA)/ Propidium iodide (PI) | **Microscopic approach**  CFDA is metabolised by living cells’ esterase activity [8], resulting in fluorescein fluorescence [9]; PI passes the membrane of dead cells and stains DNA [10]. | - A reliable and fast method - Precise viability determination due to two dyes | - Same disadvantages as MB - Fluorescence equipment necessary - Additional cause due to DNA intercalating properties of PI - Double staining required - Non-representative cell numbers |
| Carboxyfluorescein-Diacetate (CFDA) | **Flow-cytometric approach**  CFDA is metabolised by living cells, resulting in fluorescence [11]; Dead cells are unstained. | - Simultaneous determination of viability and vitality possible - High cell numbers in fast a measurement time - Distinguish between subpopulations possible | - Flow-cytometric device necessary - Expertise in equipment and data analysis required - Expensive dye and equipment maintenance |
| Colony-forming units (CFU) | **Growth assay**  Viable cells can grow in a solid or liquid medium, forming colonies. | - Precise determination of viable cells due to one formed colony per viable cell - Easy assay not requiring expert-knowledge [12] | - Time-consuming due to dependency on growth properties - Cell number determination is necessary - Dilutions necessary - Limited cell number |
| Membrane fluidity (Generalised polarisation (GP)) | **Fluorescence-based method**  Viable cells can regulate membrane fluidity by incorporation of ergosterol [13]. | - Easy and fast measurement due to the independency of cell number - No expert knowledge required - Mean value of representative cell number - Transferability to other laboratories due to independency of specific equipment | - Fluorescence microscope or photometer [13]/ multi-plate reader necessary - Alternatively: Fluorescence anisotropy [14] - Less literature data present for comparison or data interpretation - Very sensitive due to temperature effects [15] |

**Results:**

TEM measurements resulted in a single-particle diameter of 12.72 ± 3.83 nm (Figure S2 A). The BIONs used for experiments agglomerate up to 2770 ± 529 nm (Figure S2 B) and have a zeta potential of −24.98 ± 2.07 mV in the corresponding buffer for the 1 g L^−1^ solution. Similar behaviour was observed for the 0.1 g L^−1^ s solution, where the hydrodynamic diameter is 2037 ± 149 nm, and the zeta potential is measured to be −21.73 ± 0.75 mV. This data shows that the BIONs have a small primary particle diameter; however, they tend to form agglomerates in solution, as the attractive forces are dominating. These observations are consistent with those of Wittmann et al. [16], where the colloidal stability of the BIONs was investigated.

**Tables:**

**Table S2**: The impact of nanoparticles on P. pastoris cells, determined by flow cytometry and membrane fluidity; N = 3

| Theoretical viability | Measurement method | *P. pastoris* cells before nanoparticle addition [%] | *P. pastoris* cells 1 h after nanoparticle contact [%] |
| --- | --- | --- | --- |
| 50 % | Flow cytometry | 47.94 ± 5.63 | 50.97 ± 1.12 |
| 100 % | Flow cytometry | 100 ± 6.07 | 99.44 ± 0.10 |
| 50 % | Membrane fluidity | GP: -5.96 10^-5^ ± 0.04  50.54 ± 11.35 | GP: -1.10 10^-2^ ± 0.02  47.21 ± 4.85 |
| 100 % | Membrane fluidity | GP: 0.46 ± 0.02  101.04 ± 6.11 | GP: 0.42 ± 0.03  91.38 ± 9.01 |

**Figures:**

**Figure S1:** Comparison of the assay methylene-blue, Mg-ANS, CFDA and cfu with industrial yeast samples of Saccharomyces ssp. pastorianus in an anaerobic fermentation. Microscopic approaches: 130-317 cells per sample; CFDA: 20000 cells per sample; cfu: 59-157 counted colonies per sample; N=3

| A | B |
| --- | --- |
| 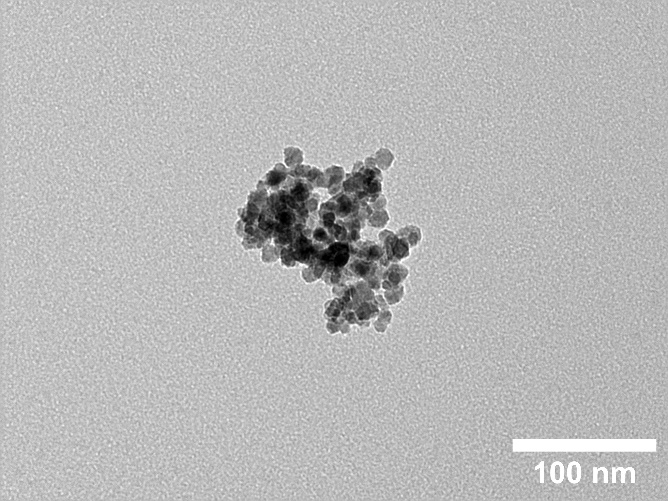 | 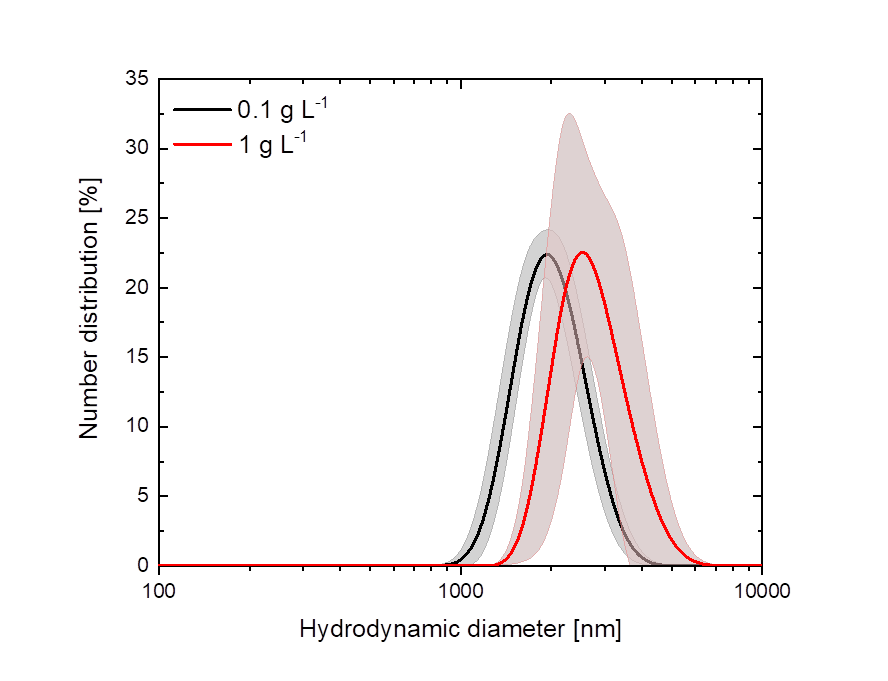 |

**Figure S2:** A: TEM image of BIONs. B: Number distribution of 1 g L^−1^ (red) and 0.1 g L^−1^ (black) BION solution in 20 mM MOPS pH 7.3. Average number distribution (dark) and standard deviation (transparent) of N=3 shown

**References:**

1. Smart, K.A., et al., *Use of Methylene Violet Staining Procedures to Determine Yeast Viability and Vitality.* Journal of the American Society of Brewing Chemists, 1999. **57**(1): p. 18-23.

2. Bonora, A. and D. Mares, *A simple colorimetric method for detecting cell viability in cultures of eukaryotic microorganisms.* Current Microbiology, 1982. **7**(4): p. 217-221.

3. Painting, K. and B. Kirsop, *A quick method for estimating the percentage of viable cells in a yeast population, using methylene blue staining.* World Journal of Microbiology and Biotechnology, 1990. **6**(3): p. 346-347.

4. O'Connor-Cox, E., et al., *Methylene blue staining: Use at your own risk.* Master Brew Assoc Am Tech Q, 1997. **34**: p. 306-312.

5. Land, M., et al., *Citrate-Buffered Methylene Violet Stain as an Alternative to Conventional Stains Used to Determine Yeast Viability.* Journal of the American Society of Brewing Chemists, 2001. **59**(4): p. 232-233.

6. White, L.R., et al., *Comparison of Yeast Viability/Vitality Methods and Their Relationship to Fermentation Performance*, in *Brewing Yeast Fermentation Performance*. 2003. p. 138-148.

7. McCaig, R., *Evaluation of the Fluorescent Dye 1-Anilino-8-Naphthalene Sulfonic Acid for Yeast Viability Determination.* Journal of the American Society of Brewing Chemists, 1990. **48**(1): p. 22-25.

8. Saruyama, N., et al., *Quantification of metabolic activity of cultured plant cells by vital staining with fluorescein diacetate.* Analytical Biochemistry, 2013. **441**(1): p. 58-62.

9. Breeuwer, P., et al., *Characterization of uptake and hydrolysis of fluorescein diacetate and carboxyfluorescein diacetate by intracellular esterases in Saccharomyces cerevisiae, which result in accumulation of fluorescent product.* Applied and environmental microbiology, 1995. **61**(4): p. 1614-1619.

10. Kolek, J., et al., *Evaluation of viability, metabolic activity and spore quantity in clostridial cultures during ABE fermentation.* FEMS Microbiology Letters, 2016. **363**(6).

11. Weigert, C., et al., *Application of a short intracellular pH method to flow cytometry for determining Saccharomyces cerevisiae vitality.* Applied and Environmental Microbiology, 2009. **75**(17): p. 5615-5620.

12. Hung, C.-W., et al., *A simple and inexpensive quantitative technique for determining chemical sensitivity in Saccharomyces cerevisiae.* Scientific reports, 2018. **8**(1): p. 11919-11919.

13. Learmonth, R.P. and E. Gratton, *Assessment of Membrane Fluidity in Individual Yeast Cells by Laurdan Generalised Polarisation and Multi-photon Scanning Fluorescence Microscopy*, in *Fluorescence Spectroscopy, Imaging and Probes: New Tools in Chemical, Physical and Life Sciences*, R. Kraayenhof, A.J.W.G. Visser, and H.C. Gerritsen, Editors. 2002, Springer Berlin Heidelberg: Berlin, Heidelberg. p. 241-252.

14. Swan, T.M. and K. Watson, *Membrane fatty acid composition and membrane fluidity as parameters of stress tolerance in yeast*. Vol. 43. 1997. 70-7.

15. Leach, M.D. and L.E. Cowen, *Membrane fluidity and temperature sensing are coupled via circuitry comprised of Ole1, Rsp5, and Hsf1 in Candida albicans.* Eukaryotic cell, 2014. **13**(8): p. 1077-1084.

16. Wittmann, L., C. Turrina, and S.P. Schwaminger, *The Effect of pH and Viscosity on Magnetophoretic Separation of Iron Oxide Nanoparticles.* Magnetochemistry, 2021. **7**(6): p. 80.
